# Hybrid Deep Recurrent Neural Networks for Noise Reduction of MEMS-IMU with Static and Dynamic Conditions

**DOI:** 10.3390/mi12020214

**Published:** 2021-02-20

**Authors:** Shipeng Han, Zhen Meng, Xingcheng Zhang, Yuepeng Yan

**Affiliations:** 1 Institute of Microelectronics, Chinese Academy of Sciences, Beijing 100029, China; hanshipeng@ime.ac.cn (S.H.); zhangxingcheng@ime.ac.cn (X.Z.); yanyuepeng@ime.ac.cn (Y.Y.); 2 University of Chinese Academy of Sciences, Beijing 100049, China

**Keywords:** recurrent neural network (RNN), long short term memory (LSTM), gated recurrent unit (GRU), MEMS gyroscope, noise reduction

## Abstract

Micro-electro-mechanical system inertial measurement unit (MEMS-IMU), a core component in many navigation systems, directly determines the accuracy of inertial navigation system; however, MEMS-IMU system is often affected by various factors such as environmental noise, electronic noise, mechanical noise and manufacturing error. These can seriously affect the application of MEMS-IMU used in different fields. Focus has been on MEMS gyro since it is an essential and, yet, complex sensor in MEMS-IMU which is very sensitive to noises and errors from the random sources. In this study, recurrent neural networks are hybridized in four different ways for noise reduction and accuracy improvement in MEMS gyro. These are two-layer homogenous recurrent networks built on long short term memory (LSTM-LSTM) and gated recurrent unit (GRU-GRU), respectively; and another two-layer but heterogeneous deep networks built on long short term memory-gated recurrent unit (LSTM-GRU) and a gated recurrent unit-long short term memory (GRU-LSTM). Practical implementation with static and dynamic experiments was carried out for a custom MEMS-IMU to validate the proposed networks, and the results show that GRU-LSTM seems to be overfitting large amount data testing for three-dimensional axis gyro in the static test. However, for *X*-axis and *Y*-axis gyro, LSTM-GRU had the best noise reduction effect with over 90% improvement in the three axes. For *Z*-axis gyroscope, LSTM-GRU performed better than LSTM-LSTM and GRU-GRU in quantization noise and angular random walk, while LSTM-LSTM shows better improvement than both GRU-GRU and LSTM-GRU networks in terms of zero bias stability. In the dynamic experiments, the Hilbert spectrum carried out revealed that time-frequency energy of the LSTM-LSTM, GRU-GRU, and GRU-LSTM denoising are higher compared to LSTM-GRU in terms of the whole frequency domain. Similarly, Allan variance analysis also shows that LSTM-GRU has a better denoising effect than the other networks in the dynamic experiments. Overall, the experimental results demonstrate the effectiveness of deep learning algorithms in MEMS gyro noise reduction, among which LSTM-GRU network shows the best noise reduction effect and great potential for application in the MEMS gyroscope area.

## 1. Introduction

MEMS-IMU has attracted much attention in the recent years owing to their low cost, small size, and ease of integration [1]. This device is ushering in a huge market demand, as it has become an important component of different navigation systems, attitude control devices [2], unmanned aerial vehicles [3], robot navigation [4,5,6], satellite systems [7], etc. MEMS-IMU system is often affected by various factors characterized on environmental, electronic, and manufacturing noises all leading to random navigation errors when using MEMS-IMU [8,9]. These random errors reduce the accuracy of MEMS-IMUs and as well limit their applications. Basically, MEMS-IMU consists of three orthogonal MEMS gyroscopes and MEMS accelerometers which are sensors used to determine navigation accuracy in MEMS-IMU. Therefore, the effective mode tuning and noise reduction of MEMS gyroscope is the key to improve the accuracy of MEMS-IMU system.

Mode tuning (or mode matching) is utilized for minimizing the frequency difference between the drive and detection modes in order to improve the sensitivity of the gyro and to achieve a highly accurate measurement [10]. Typically, two rings of automatic gain control modulates the drive mode of the gyro and phase locked loop to achieve a constant amplitude of drive mode vibration and locking of resonant frequency, while the detection mode is balanced by two rings of force to eliminate the Coriolis and quadrature forces [11]. In order to resist external disturbances, increase the robustness of the system, the resonant frequency of the drive, and sense mode of the gyro need to be matched equally. In Ref. [11], the matching of the drive and sense mode is achieved by generating DC voltage through an additional conditioner, which is applied to the adjustable electrostatic unit and the quadrature correction unit respectively. In Ref. [12], the frequency difference is first adjusted manually, with a genetic algorithm utilized to further adjust the frequency difference to a minimum value. Additionally, the Refs. [13,14,15,16] is based on the amplitude of the quadrature error reaching its maximum value during mode matching. Furthermore, the bandwidth is controlled by varying the tuning voltage such that the frequency separation of the drive and detection resonant modes is controllable, and thus gyroscopic accuracy improvement is achieved. Mode-matching control can also be achieved by exploiting the phase relationship between the drive signals and the redundant quadrature error [17], and by the mode-matching method with an applied electrostatic force phase frequency characteristic to achieve accuracy improvement [18]. Considering the coupling errors caused by external disturbances in ring and axisymmetric resonators, methods derived from ring dynamics [19] can be used to tune the resonator mode coupling [20,21], and similar techniques can be used to tune quadrupole gyros [22] and the parasitic coupling in MEMS resonators [23]. In addition, some advanced control algorithms have been utilized to improve the drive control performance. In Ref. [24], dynamic mode manifold and super-twisting sliding mode are introduced to improve the performance of MEMS gyroscopes. For some coupling errors caused by asymmetric structures and fabrication error, fuzzy sliding mode control in [25] and adaptive sliding mode control in [26] are employed to improve its robustness.

In addition to mode tuning, more and more works on various schemes for noise reduction and compensation algorithms have been proposed to improve the accuracy of MEMS gyroscope. In the last decade, many representative methods emerged for denoising MEMS gyro. These include autoregressive sliding average [27], Allan variance [28], Kalman filtering [29], wavelet thresholding [30], and machine learning represented by neural network (NN) and support vector machine (SVM) [31,32,33,34]. Since the output signal of MEMS gyro is generally non-stationary, the original signal needs to be smoothed. The smoothing process entails the extraction of a stable random drift sequence via period-based analysis, with linear autoregressive, sliding average, or mixed autoregressive sliding average utilized in fitting the random drift sequence to obtain a smooth signal. The main operations in autoregressive averaging model are the determination of suitable model structure, identification of the model parameters, and validation of the model’s applicability [27]. The Allan variance method is a standard analytical approach for describing and identifying the various error sources in a MEMS gyroscope and similarly for statistical characterization of the noise source [35]. Kalman Filtering is based on the known statistical properties of the external disturbance signal and dynamics model of the system. However, model and noise statistics are unknown in most cases of real systems. Thus, the opinion of designing the Kalman Filter due to the presence of inaccurate model and noise statistics can lead to optimality loss, great reduction in estimation accuracy, and filter divergence. To solve the problem of Kalman Filter divergence, various improved adaptive Kalman Filters have been developed in recent years to deal with measurement uncertainties [36]. Wavelet thresholding is a common method for denoising MEMS gyro output signals, and it has the characteristics of multiresolution analysis. By decomposing the signal in multiple scales and levels, the details of the signal can be observed in a time-frequency variable window, which has obvious advantages in the analysis of non-smooth signals. By taking advantage of wavelet thresholding, transition errors, and high-frequency noise of low-frequency signals can be effectively eliminated, while fast and accurate initial calibration can be achieved [37]. NN has the ability to learn from the useful signals of the original data, but it cannot learn from the noisy components, and therefore are usually used for signal denoising or error compensation [38]. In Ref. [39], a NN-based denoising model is proposed to suppress high noise components, which is essential for optimizing prefiltering methods. Radial basis function neural network is also suitable for compensation of random drift of MEMS gyroscopes due to their nonlinear, adaptive, and self-learning characteristics [40]. The SVM method was initially used for classification but with the introduction of insensitive loss functions, SVM has successfully been extended for regression estimation of nonlinear systems. SVM is applicable for nonlinear processing, thus it is widely used in MEMS sensors error compensation [32].

For predictive processing of non-stationary signals of time series, recurrent neural network (RNN) seems to perform well. RNN is a network with memory function. It manifests itself in the form that the network keeps the previous information and applies it to the computation of the current output. Specifically, the nodes between the hidden layers are connected, and the input of the hidden layers includes not only the output of the input layer but also the output of the hidden layer at the previous moment [41]. Theoretically, RNNs are capable of processing sequence data of any length, thus are best suited to solving problems with continuous sequences and are good at learning patterns from sample-to-sample with some sequential meaning [42]. RNNs have been successfully applied for natural language processing, speech recognition, web content recommendation, etc. [43,44]. In recent years, RNN technology is also being applied for solving problems such as signal correction and error compensation in MEMS sensors. A new method for real-time estimation and compensation of random drift of MEMS gyroscopes is proposed by combining trace-free Kalman filter and RNN in [45]. Results from the experimental study show the method to be effective and superior. Although RNN is not cost effective for time series signal processing, it is prone to gradient disappearance and gradient explosion due to a small memory value [46]. Hence, long short term memory (LSTM) and gated recurrent unit (GRU) are two improved algorithms of RNN that have been developed to solve these problems [47,48]. The application of RNN for MEMS sensor signal processing is just emerging with relatively little research undertaken so far. In addition, in Ref. [49], LSTM was used to denoise the output signal of MEMS gyroscope while only two minutes gyroscope static data were used for model testing. Nevertheless, results show the method is effective for improving accuracy of MEMS gyroscope. The GRU approach does not only solve the gradient disappearance and gradient explosion problems of RNN, but also utilized fewer parameters than its LSTM counterpart. Thus, GRU has a greatly reduced training time, which could make it suitable for processing time series data [48]. In Ref. [50], both GRU and LSTM are mixed for MEMS gyro noise suppression. However, the study only used static data for training and prediction, and the training loss and standard deviation from the training data cannot be used for intuitive or quantitative analysis. Therefore, the performance of both LSTM and GRU in MEMS gyroscope denoising still requires further investigation, and the effectiveness of LSTM and GRU needs to be fully quantified and analyzed from static and dynamic experimental perspectives. Inspired by these works, four hybrid modes of RNN models, including LSTM-LSTM, GRU-GRU, LSTM-GRU, and GRU-LSTM, are proposed for noise reduction in a customized MEMS-IMU developed in our laboratory. Model validation were achieved by acquiring twelve minutes static and dynamic data for network training and testing, while the corresponding results were quantitatively analyzed to systematically evaluate the algorithms.

This study aims to develop hybrid modes of deep learning models for noise reduction of MEMS gyroscope in different motion conditions and accelerating the intelligence of MEMS gyroscope. The hybrid models will be embedded into MEMS-IMU to create a promising way of improving accuracy of the MEMS-IMU system. The remainder of this paper proceeds as follows. Section 2 describes the mathematic principle of the LSTM and GRU, and their hybrid modes. Section 3 introduces the experimental platform construction, data acquisition, parameter determination, static and dynamic data prediction results, and quantitative analysis results of the four algorithms. Section 4 summarizes the results of this paper and provides an outlook for future work.

## 2. Methods

In comparison with convolutional neural networks, RNN has the capability of handling sequential data. Although, RNNs are hard to train as they have difficulty in handling long term dependencies in practical applications. The improved versions, i.e., LSTM and GRU, have been developed and successfully applied to mitigate the limitations of the primitive RNN, especially the problems of long-term memory and computation time [47,48,51]. In this section, dual layered recurrent networks built on LSTM and GRU are developed for noise identification and elimination in a MEMS-IMU device were proposed. We followed an in-depth design analysis of the networks to simplify the networks’ modeling complexities. For this, working principles of LSTM and GRU were given into some details using mathematical and schematic illustrations. Then, details of the homogenous and heterogeneous hybrid networks formed on the conventional LSTM and GRU models were discussed.

### 2.1. The Principle of LSTM

**Figure 1:**
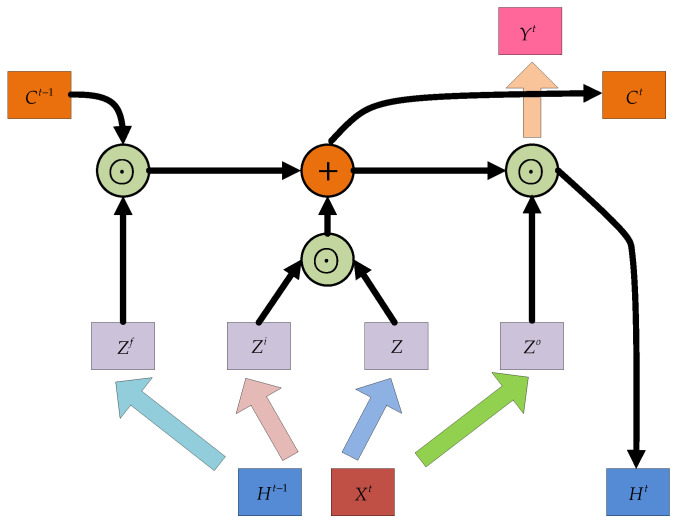
The structure of long short term memory (LSTM).

LSTM is a special kind of RNN designed to solve the gradient disappearance and explosion problems when training of long sequences of data. Compared with conventional RNN, LSTM models do produce better generalization and prediction performances in longer sequences. Thus, this type of recurrent network holds great prospects for noise suppression in time series signals of the MEMS gyroscope. Following the principle of [52,53], which is presented in Figure 1, LSTM model, mainly has an algorithmic processor (called “cell”) that can be used to determine whether information contained in a sequence data is useful or otherwise. In addition to the cell states, the LSTM introduces three gating structures, namely the input gate, the forget gate and the output gate. These gates allow information to pass selectively in an attempt that the LSTM structure do protect and control information. As opposed to what is found in many related literature, we introduce more details about the three gates. Unlike RNN that has only one transfer state, LSTM has two states, which are the cell state $C^{t}$ and hidden state $H^{t}$. In general, the output $C^{t}$ is the $C^{t-1}$ transferred from the previous state plus some values, while the $H^{t}$ often has great variations under different nodes.

The forget gate is a certain probability control node that is used to decide whether to forget the hidden cell state from a previous layer or not. For the current input, there is the hidden state $H^{t-1}$ of the previous sequence and the input data $X^{t}$ of the current sequence, and then the output of $Z^{f}$ the forgetting gate is obtained by a sigmoid activation function. Since the output $Z^{f}$ of sigmoid is between [0, 1], it indicates the weight to let the corresponding information pass. A value of “0” means “don’t let any message pass”, and oppositely “1” means “let all messages pass”. The mathematical expression is as Equation (1). Where σ(⋅) is the sigmoid function, $W^{f}$ is the weight matrix of forget gate, $\left[H^{t-1},X^{t}\right]$ means connecting two vectors into a longer vector, and $B^{f}$ is the bias of the forget gate.

(1) $$Z^{f}=\sigma (W^{f}\cdot [H^{t-1},X^{t}]+B^{f})$$

This stage of the input gate focuses on the selective memorization of the input $X^{t}$. The structure of Figure 1 shows that the input gate consists of two parts with the first part using the sigmoid activation function to decide which information needs to be updated. In addition, information selection is controlled by the input gating signal $Z^{i}$. Similarly, the second part of the structure uses the *tanh* activation function, on the current input information, which also is the previously calculated cell state Z. The updated cell state $C^{t}$ consists of two parts given in Equations (2)–(4): the first part is the product of $C^{t-1}$ and the output $Z^{f}$ of the forgetting gate, and the second part is the product of input gate $Z^{i}$ and Z. Where σ(⋅) is a sigmoid function,$W^{i}$ and $B^{i}$ are the weight matrix and bias of the input gate, respectively. $\left[H^{t-1},X^{t}\right]$ means connecting two vectors into a longer vector. $W^{c}$ and $B^{c}$ are the weight matrix and bias of cell state, respectively. ⊙ is the Hadamard product.

(2) $$Z^{i}=\sigma (W^{i}\cdot [H^{t-1},X^{t}]+B^{i})$$

(3) $$Z=\tanh(W^{c}\cdot [H^{t-1},X^{t}]+B^{c})$$

(4) $$C^{t}=Z^{f}⊙C^{t-1}+Z^{i}⊙Z$$

The output gate is used to control how much information from the cell state $C^{t}$ is output to the current output value $H^{t}$. This stage is also controlled by a sigmoid function that determines which parts of the cell state are to be output, which is called $Z^{o}$. Then, the part of the cell state to be output is processed by *tanh* to a value of [−1, 1] and multiplied with the output of the sigmoid gate to achieve a definite output of the current $H^{t}$. Similar to ordinary RNN, the output $Y^{t}$ is often obtained by changing $H^{t}$. Where σ(⋅) is a sigmoid function, $W^{o}$ and $B^{o}$ are the weight matrix and bias of output gate, respectively. $\left[H^{t-1},X^{t}\right]$ means connecting two vectors into a longer vector. ⊙ is the Hadamard product, and $W^{\prime }$ is the corresponding weight matrix.

(5) $$Z^{o}=\sigma (W^{o}\cdot [H^{t-1},X^{t}]+B^{o})$$

(6) $$H^{t}=Z^{o}⊙\tanh(C^{t})$$

(7) $$Y^{t}=\sigma (W^{\prime }H^{t})$$

### 2.2. The Principle of GRU

**Figure 2:**
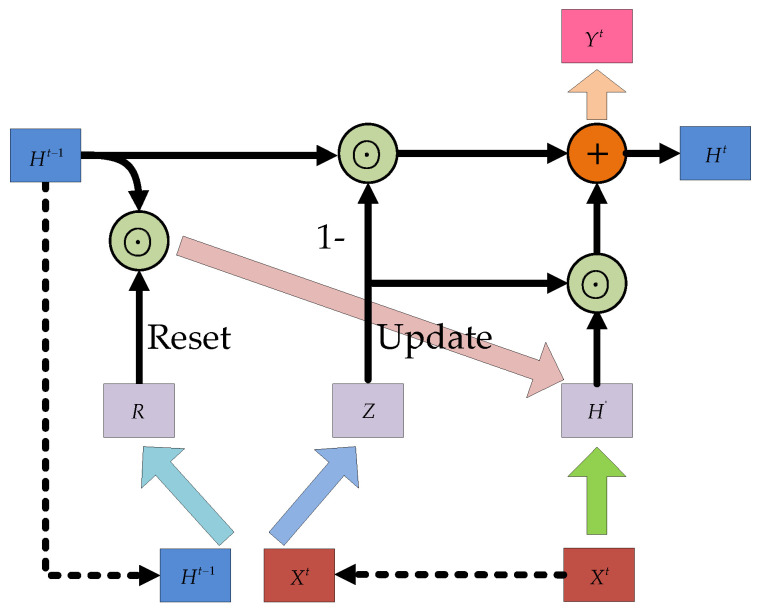
The structure of gated recurrent unit (GRU).

As a variant of LSTM, GRU has been developed and shown to produce correspondingly competent results that were similar to LSTM models. GRUs, proposed by Chung in 2014, only differed from LSTMs in how their gates monitor information flow from erstwhile time steps while the gating mechanisms in LSTMs rather control the flow of information within internal cell unit [48]. GRUs are often preferred for solving problems related to long-term memory and gradient in backpropagation as they can achieve comparable results as LSTM. Further, GRUs are comparably easier to train and provides improved training efficiency. GRU, similar to LSTM, also controls the information flow by “gate”, but with one less gate than LSTM and without cell states. A detailed GRU structure introduced in [52,54] is analyzed as shown in Figure 2, and this is considered for implementation in this study. The input and output structure of GRU is the same as that of a normal RNN. There is a current input $X^{t}$, and a hidden state $H^{t-1}$ passed down from the previous node, which contains the relevant information of the previous node. Combining $X^{t}$ and $H^{t-1}$, GRU gets the output $Y^{t}$ of the current hidden node and the hidden state $H^{t}$ passed to the next node. GRU, similar to LSTM, also controls the information flow by “gate”, but with one less gate than LSTM and also without cell states. 

According to the principle of GRU, there are two gates in the GRU structure, namely the reset gate and the update gate, and both gates are determined by the state $H^{t-1}$ of the previous transmission down and the input $X^{t}$ of the current node. They can be used the Equations (8) and (9) to show the relationship.

(8) $$R=\sigma (W^{r}\cdot [H^{t-1},X^{t}])$$

(9) $$Z=\sigma (W^{z}\cdot [H^{t-1},X^{t}])$$

where R stands for the reset gate, Z represents the update gate, and σ(⋅) is a sigmoid function that transforms the data into a value in the range of 0–1, thus acting as a gating signal. $W^{r}$ and $W^{z}$ is the weight matrix of reset gate and update gate, respectively, while $\left[H^{t-1},X^{t}\right]$ is an operation that joins two vectors. After obtaining the gating signal, the reset gate is used to get the “reset” data $H^{t-1\prime }$, then $H^{t-1\prime }$ is spliced with the input $X^{t}$, and finally the data is deflated to [−1,1] by the *tanh* activation process in Equations (10) and (11).

(10) $$H^{t-1\prime }=H^{t-1}⊙R$$

(11) $$H^{\prime }=\tanh(W\cdot [H^{t-1\prime },X^{t}])$$

where ⊙ is the Hadamard product of contents in the reset gate and content of the hidden node; similarly, W is the corresponding weight matrix. $H^{\prime }$ mainly contains the $X^{t}$ data of the current input. In addition, adding $H^{\prime }$ to the current hidden state in a targeted way is equivalent to remembering the current state at the moment.

Lastly, the memory state is updated by employing content of the update gate Z to achieve forgetting and selective memory functions. Gating signal Z ranges from 0 to 1 while signal values closer to the gating signal value tend to be remembered, and the ones closer to zero, tend to be “forgotten”. Where $(1-Z)⊙H^{t-1}$ means selective “forgetting” of the original hidden state, $Z⊙H^{\prime }$ indicates selective “memory” of $H^{\prime }$ containing current node information; σ(⋅) is the sigmoid function, $W^{\prime }$ is the corresponding weight matrix, and the output $Y^{t}$ is often obtained by changing $H^{t}$.

(12) $$H^{t}=(1-Z)⊙H^{t-1}+Z⊙H^{\prime }$$

(13) $$Y^{t}=\sigma (W^{\prime }H^{t})$$

### 2.3. Hybrid Modes of LSTM and GRU

**Figure 3:**
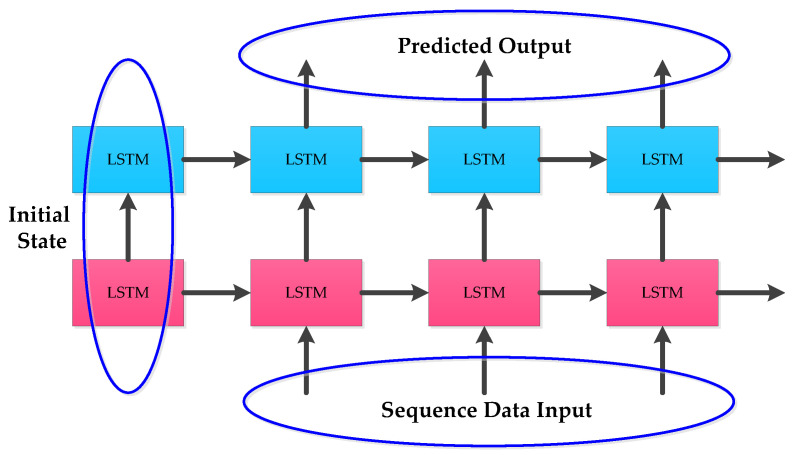
The structure of LSTM-LSTM.

**Figure 4:**
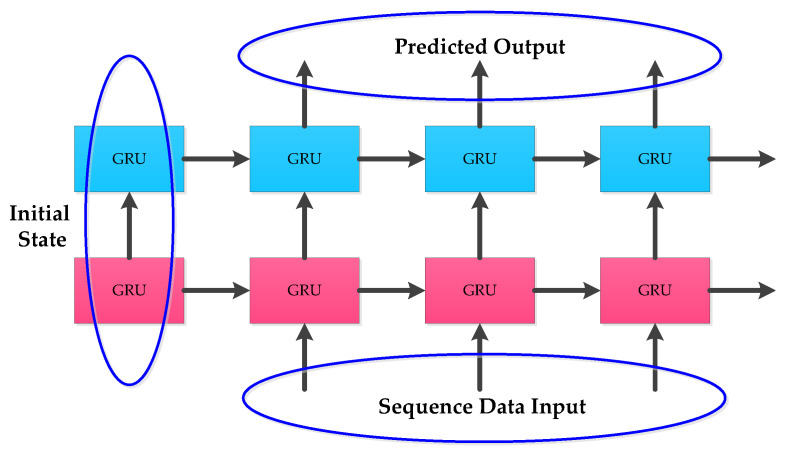
The structure of GRU-GRU.

**Figure 5:**
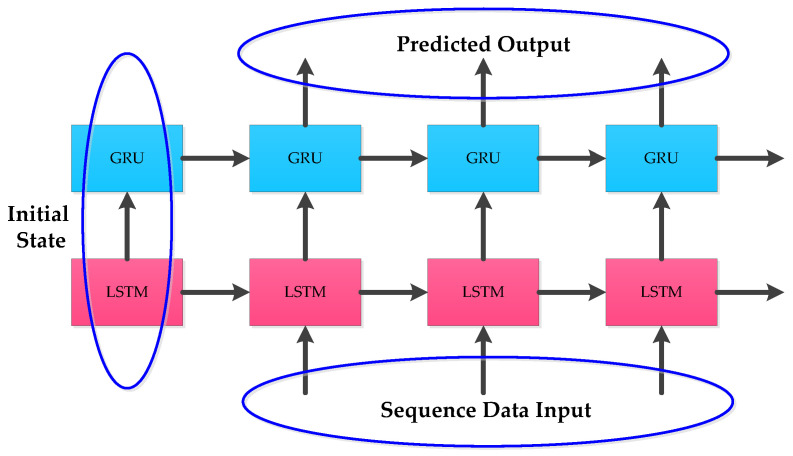
The structure of LSTM-GRU.

**Figure 6:**
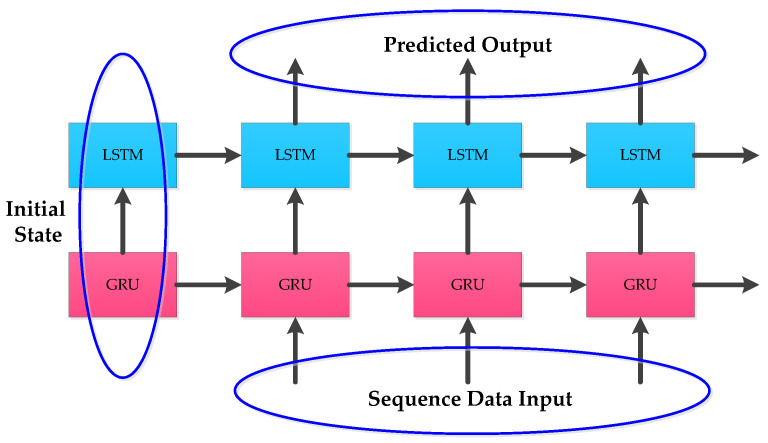
The structure of GRU-LSTM.

To solve the gradient disappearance and explosion problems of RNN, the internal structure of some RNN units are modified in LSTM and GRU to make the networks suitable for processing sequential data. The internal structures of LSTM and GRU given in Figure 1 and Figure 2 were applied. In practical applications, instead of using single-layer LSTM or GRU, multilayer LSTM or GRU is generally used however with no more than three layers. Considering the computational performance and cost, two layer hybrid modes were designed in this paper. Figure 3 and Figure 4 show two homogenous networks one with two layers of LSTM; thus tagged as LSTM-LSTM, and the other had two GRU units thus tagged as GRU-GRU, respectively. The input and output of LSTM-LSTM were determined by the LSTM sequence, while the input and output of GRU-GRU were all derived from the GRU sequence. In Figure 5, the input layer was LSTM while the output layer was GRU and thus, a heterogeneous network abbreviated as LSTM-GRU was developed, while in Figure 6, the input layer was GRU and the output layer was LSTM to produce the other heterogeneous network referred to as GRU-LSTM. The four hybrid models of deep RNN designed above all composed of four parts. The first part was an initial state where in random initialization were made, while the second part was used for feeding the sequence data as networks’ inputs. The third stage includes a collection of hidden states in each of the LSTM or GRU layer, while the last part is where the network prediction output is done. The latter can take the state output of the last step by weighing the state from all previous steps or directly averaging them to produce the output.

## 3. Experiments and Results

### 3.1. Experiment Setup

**Figure 7:**
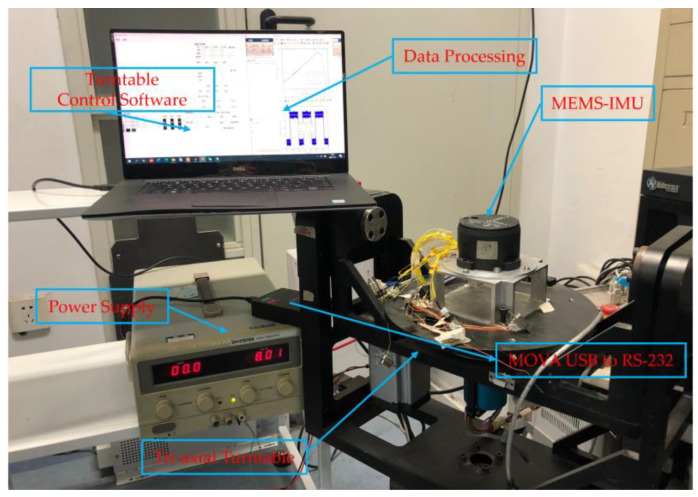
The experimental setup.

In order to verify the feasibility and effectiveness of the proposed denoising models designed above, real experimental data were collected from a custom MEMS gyroscope employed in this study. An experimental setup, shown in Figure 7, was arranged to consist of MEMS-IMU test platform. This is composed of three-orthogonal MEMS gyroscope and three-orthogonal MEMS accelerometers, tri-axial rate turntable, power supply, and a computer system installed with the data acquisition software of Microsoft visual studio 2010 and turntable controller software. In this study, raw experimental signals were acquired at the room temperature setting. The MEMS-IMU was fixed on the triaxial rate turntable, the power supply delivers 8 V and 0.12 A while the MEMS-IMU was connected. The computer system was connected to the MEMS-IMU through a MOXA USB to RS-232 data conversion cable. The computer retrieved the raw signals and stored the data via the data acquisition software.

The triaxial turntable controller was utilized to acquire a series of dynamic signals from several experiments to validate the models. The sampling frequency was set to 20 Hz for collecting the MEMS-IMU data, while the data acquisition time was approximately 700 s. In addition, our lab developed the MEMS-IMU employed in our experiment. Two types of experiments were conducted on the MEMS-IMU to evaluate the denoising performance of the proposed hybrid deep RNN models. The experiments include static and dynamic experiments of MEMS-IMU.

### 3.2. Parameters Determination

**Figure 8:**
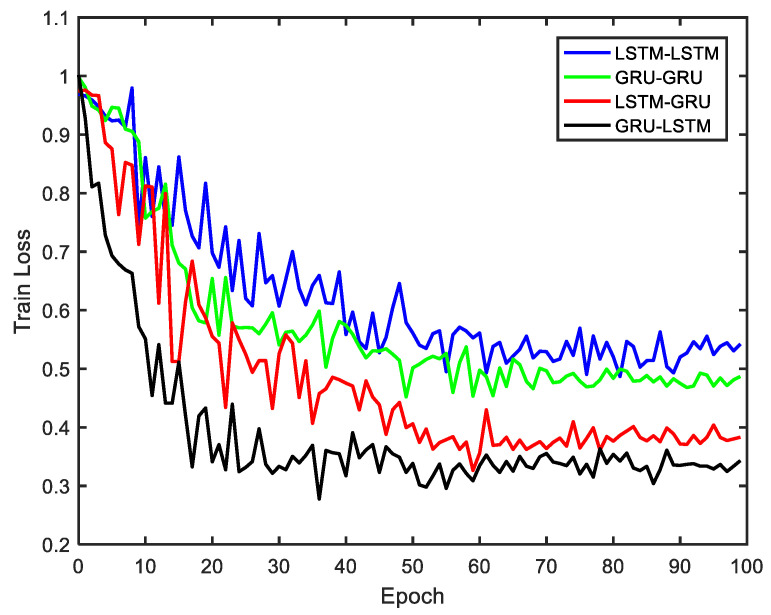
The train loss of four methods.

In order to compare and intuitively discuss the denoising performance of the four methods under the same conditions, a maximum training epoch of 100 was set based on consideration of both the training time and computer memory, while the length of the input data varied. To find the best training epoch, size of the training dataset was varied between 500 and 1500 samples, and an optimal training epoch was selected with respect to the training loss comparison performed for the four methods as shown in Figure 8. The training process shows that all the four models did not converge within 700 samples at an input data step of 100 samples. However, as the training data increased further, the GRU-LSTM model with a training data of 800 samples converged first while the other three models were yet to converge. With continual increase in the training data above 1000 samples, the remaining three methods were able to converge. These training procedures were done with the batch size set as seven, while the learning rate was set as 0.006, hidden unit was 1 and the time-step was 6. From Figure 8, the GRU-LSTM’s convergence rate was faster than those of LSTM-GRU, GRU-GRU and LSTM-LSTM; however, they all had a relatively optimal convergence epoch at 50. Taking the performance of the computer and the training time into account, a trade-off might be needed between the circulation times and the length training data. Therefore, epoch of 50 was selected as the number of iterations in the following static and dynamic data training process, to compare the noise reduction performance of the proposed four algorithms.

### 3.3. Static Experiments

**Figure 9:**
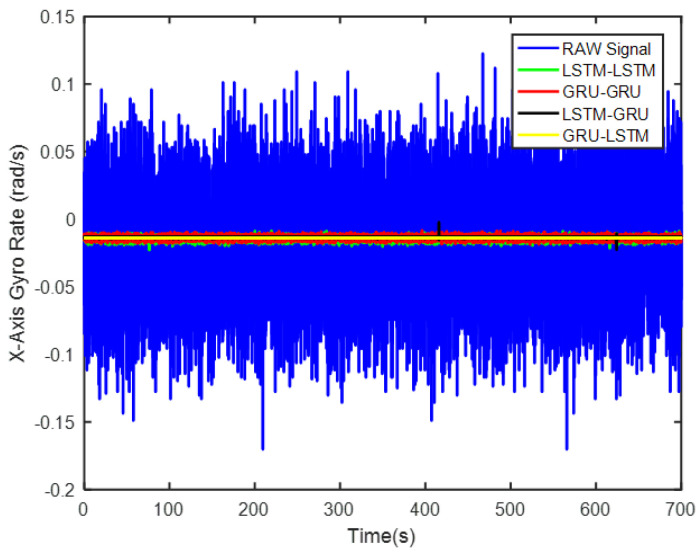
A display of the *X* axis gyro denoising results of the four methods.

**Figure 10:**
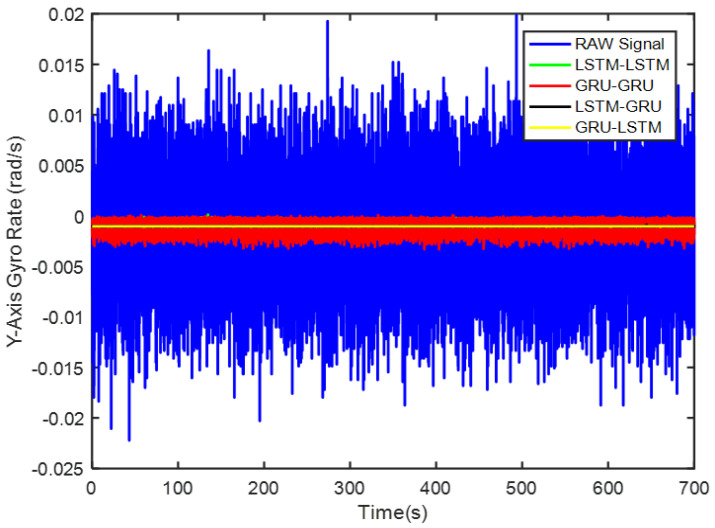
A display of the *Y* axis gyro denoising results of the four methods.

**Figure 11:**
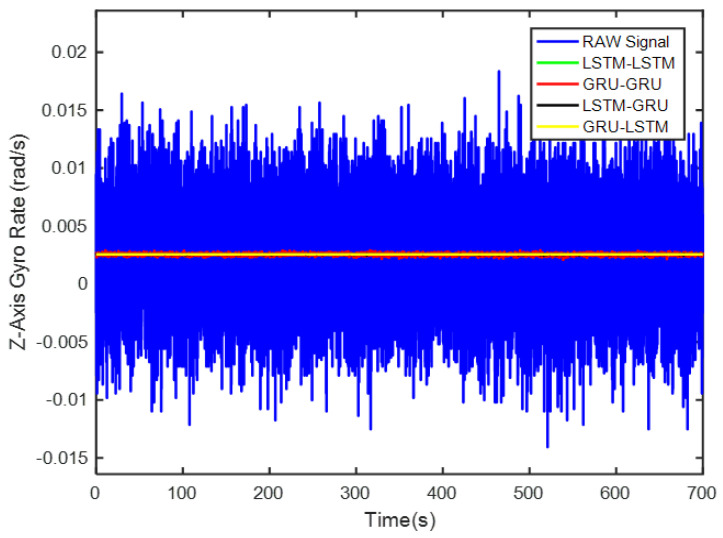
A display of the *Z* axis gyro denoising results of the four methods.

**Figure 12:**
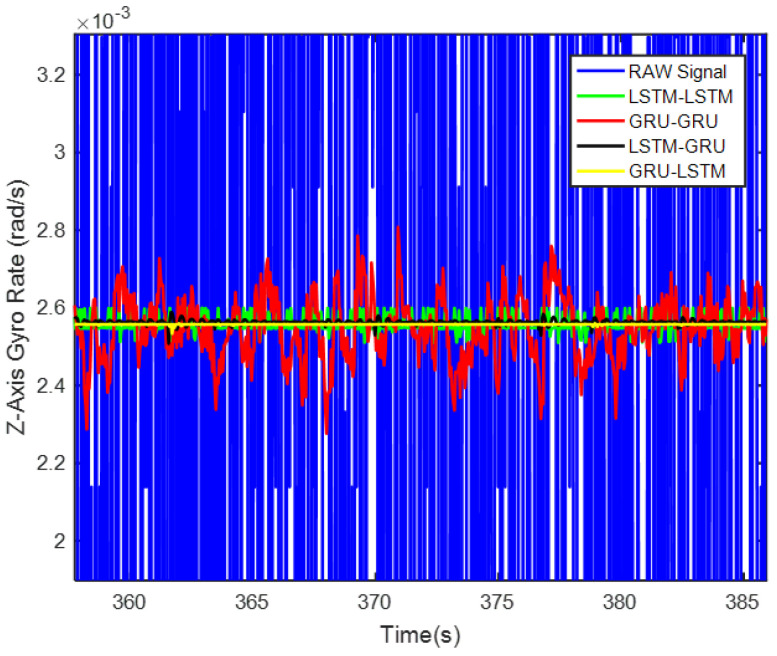
The local magnified images of denoising results in Figure 9.

**Figure 13:**
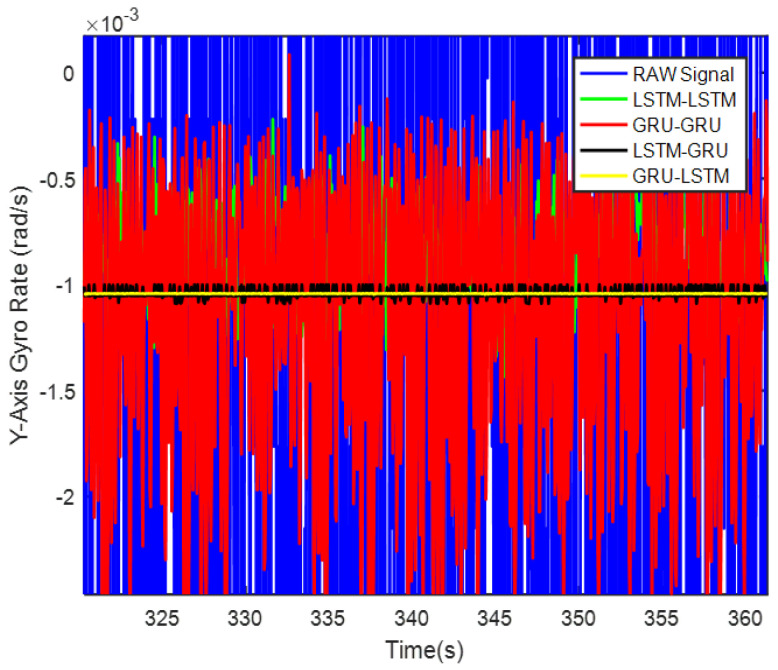
The local magnified images of denoising results in Figure 10.

**Figure 14:**
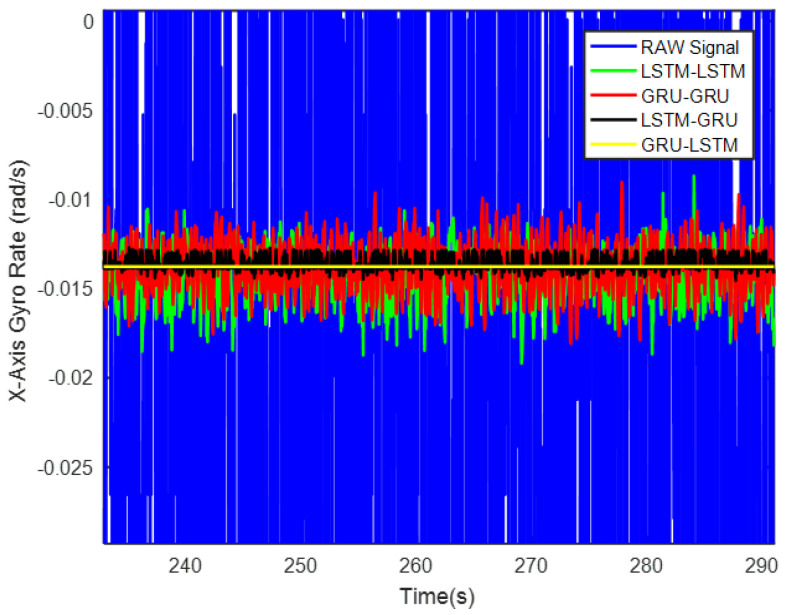
The local magnified images of denoising results in Figure 11.

In order to verify the denoising effect of the proposed algorithm under static test, the MEMS-IMU data obtained from the static experiments were first analyzed. This includes the *X*/*Y*/*Z*-axis MEMS gyros collected at room temperature for about 700 s, as shown in Figure 9, Figure 10 and Figure 11, respectively. The four algorithms proposed in this paper, LSTM-LSTM, GRU-GRU, LSTM-GRU, and GRU-LSTM, were used to denoise the gyro signals. For fair comparison between the different algorithms, all the parameters were set as determined and explained in Section 3.2. To visualize the noise reduction effect of the four algorithms, the detail part of Figure 9, Figure 10 and Figure 11 were enlarged as shown in Figure 12, Figure 13 and Figure 14. These plots show that LSTM-LSTM, GRU-GRU, and LSTM-GRU models were able to achieve significant noise reduction results for the static signals, while the denoising signal of GRU-LSTM model seemed to be applicable for large sample testing. However, to distinguish the differences between them, Allan variance was used to quantitatively analyze the noise reduction effects of each model. Allan variance is a classical time-domain analysis technique that is widely used to evaluate the performance of gyroscopes. With this, the different error coefficients of the models can be identified based on the slope of different fitted straight lines, and thus the change in performance before and after noise reduction can be determined. The Allan variance curves and the corresponding quantitative values for the denoised signals (processed form of the static signal obtained from the *X*/*Y*/*Z* axis gyro) are presented in Table 1, Table 2 and Table 3.

**Figure 15:**
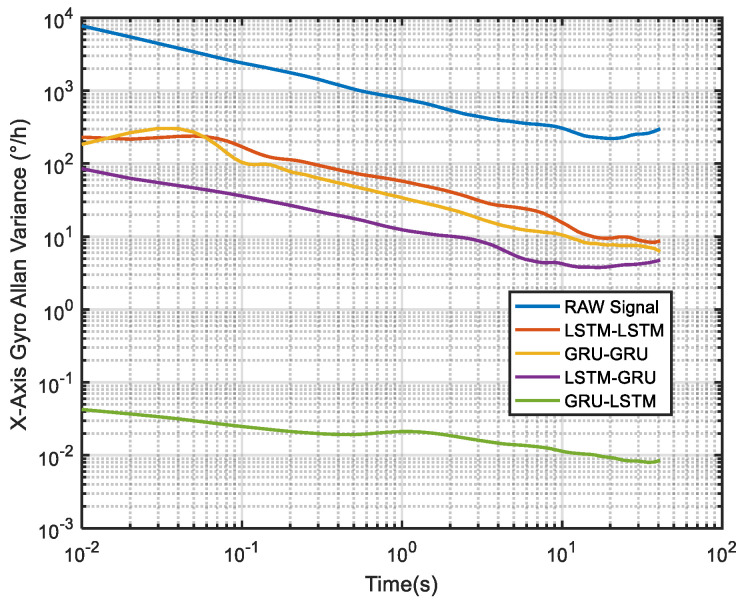
The corresponding Allan variance analysis of the *X* axis gyro.

For the *X*-axis gyro, it can be seen from Figure 15 and Table 1 that GRU-LSTM has the best performance in noise reduction, but the denoising signal appeared to be somewhat distorted (compared to others) as seen in Figure 12, such a phenomenon is most likely due to overfitting during the large amount of data testing. Therefore, the quantization noise, angular random walk, and zero bias stability parameters are not meaninglessly given in percentage. The second is the LSTM-GRU model, which also has a good noise reduction effect, with greater than 90% improvement in all three parameters. The next is the GRU-GRU model with 58%, 94%, and 64% improvement in quantization noise, angular random walk, and zero bias stability, respectively. Finally, LSTM-LSTM, although somewhat inferior to the LSTM-GRU and GRU-GRU algorithms, also shows good noise reduction, with improvements of about 48%, 92%, and 40% in quantization noise, angular random walk, and zero bias stability, respectively.

**Figure 16:**
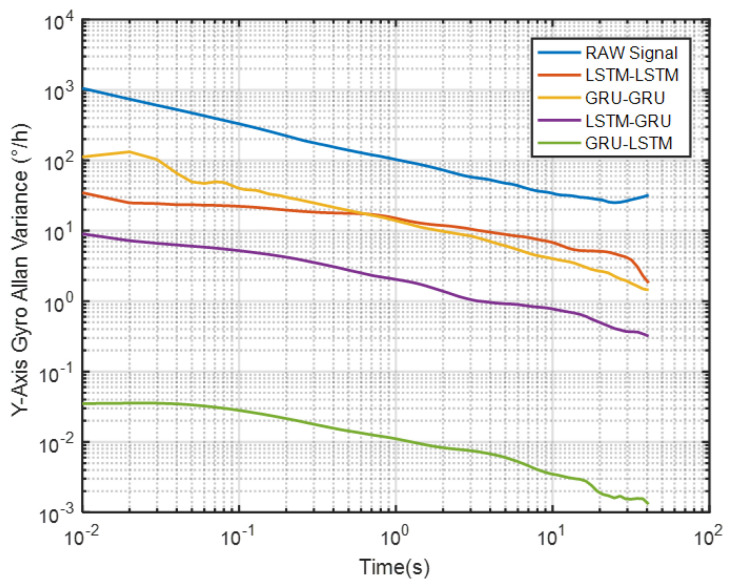
The corresponding Allan variance analysis of the *Y* axis gyro.

Again, although it can be seen from Figure 16 and Table 2 that GRU-LSTM has the best performance in noise reduction for the *Y*-axis gyro, the denoising signal also seems to be a bit distorted as seen in Figure 13. Therefore, the parameter improvement of the GRU-LSTM after denoising the signal was not given in a percentage relative to the quantization noise, angular random drift, and zero bias stabilization parameters of the original signal. Similarly, this is followed by the LSTM-GRU model with at least 90% improvement in the three parameters, while the LSTM-LSTM had relatively high performance that what was obtained for the *X*-axis gyro data. These include 77%, 96%, and 65% improvements in quantization noise, angular random walk, and zero bias stability, respectively. Lastly, the GRU-GRU again had the least performance at 82% and 47% improvement in the angular random walk and zero bias stability parameters, respectively. Surprisingly, the quantization noise had increased by 77% unlike what was observed for the *X*-axis gyro data.

**Figure 17:**
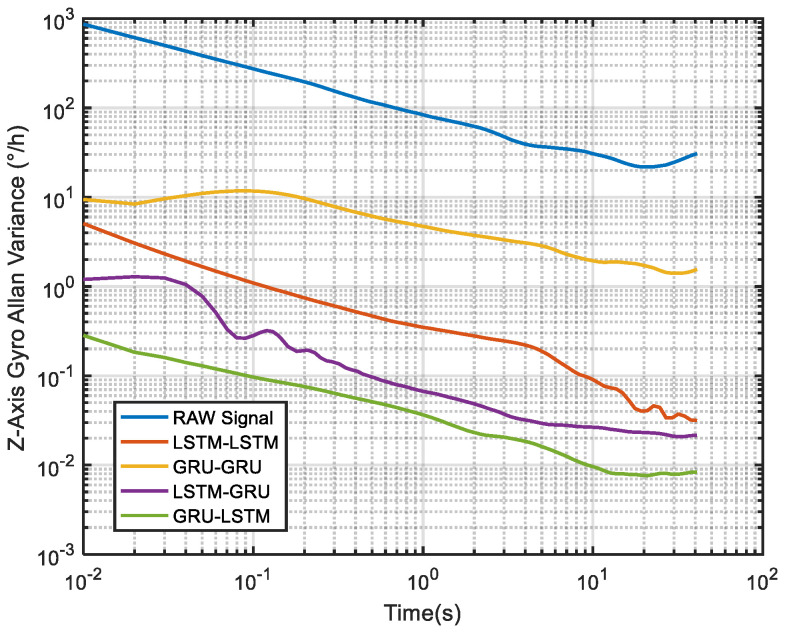
The corresponding Allan variance analysis of the *Z* axis gyro.

Lastly for the Z-axis gyro, the best noise reduction method seemed to be GRU-LSTM according to Figure 17 and Table 3, but the noise reduction signal still was also distorted as seen in Figure 14. Therefore, the improvement of three parameters was still not meaningful. However, for quantization noise and angular random walk, LSTM-GRU had better boost than LSTM-LSTM and GRU-GRU, while LSTM-LSTM shows better enhancement in zero bias stability than GRU-GRU and LSTM-GRU. Although the *X*/*Y*/*Z* axis MEMS gyroscopes were manufactured by the same MEMS batch process, there were inevitably fabrication errors and electronic signal readout errors, so the hybrid deep learning models had different enhancement accuracies.

**Figure 18:**
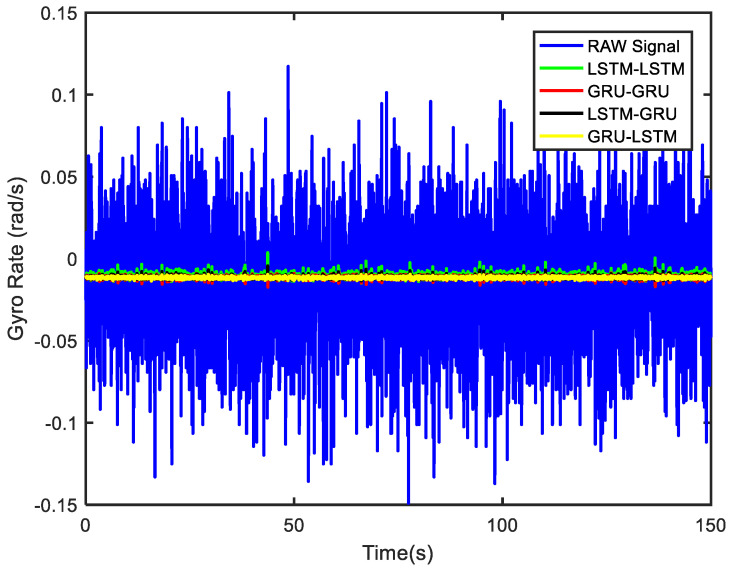
The gyro denoising results of four methods for a small sample.

**Figure 19:**
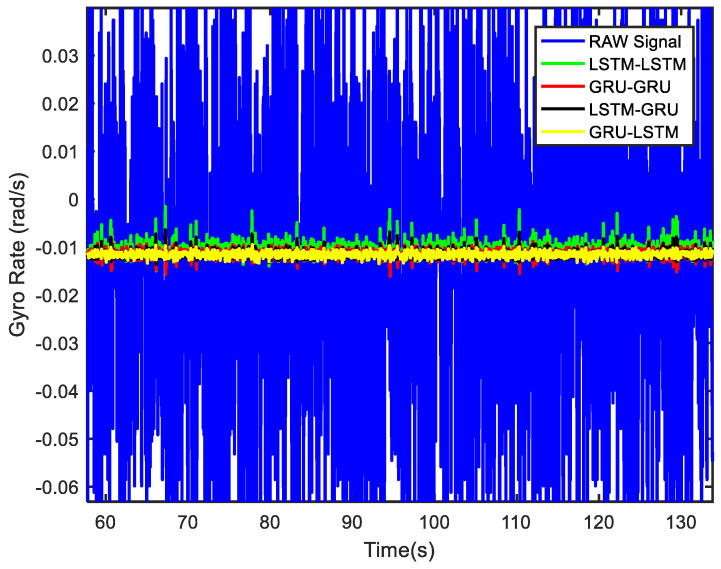
The local magnified image of denoising results in Figure 18.

According to the preliminary static experiments in Figure 12, Figure 13 and Figure 14, the denoised signal of GRU-LSTM seemed to be overfitting, and the reason for this phenomenon is most likely that the sample data is too large. To further verify whether the GRU-LSTM is effective for gyroscope signal noise reduction, a small sample data of 150 s was used to test the GRU-LSTM. The various parameters of the training and prediction data were consistent with those of the static tests above, and the only difference was the predicted data length. This experiment was respectively conducted for samples of 50, 100, and 150 s, and it was found that all four methods had good denoising results. The denoising results of the four algorithms for 150 s are shown in Figure 18, and in order to see clearly the denoising effect of different algorithms obtained in Figure 19. It can be clearly seen that all algorithms, especially GRU-LSTM, did not distort the denoised signals as in the previous experiments, but instead show the best denoising effect. The standard deviations of the signals before and after denoising in Figure 18 are shown in Table 4. As compared to the original signal, the standard deviation of the LSTM-LSTM, GRU-GRU, LSTM-GRU, and GRU-LSTM denoised signals were improved by approximately 96%, 97.5%, 97.8%, and 98.4%, respectively. 

The comprehensive analysis of the above experiments concludes that LSTM-LSTM, GRU-GRU, and LSTM-GRU had good results in the field of gyro noise reduction. In particular, LSTM-GRU was relatively superior under different sample lengths, while GRU-LSTM will show overfitting phenomenon under large samples, and small sample noise reduction effect was acceptable. However, considering the actual application scenario, MEMS gyro static output data volume were very large, so LSTM-LSTM, GRU-GRU, and LSTM-GRU were more suitable for the application in the MEMS gyro field.

### 3.4. Dynamic Experiments

**Figure 20:**
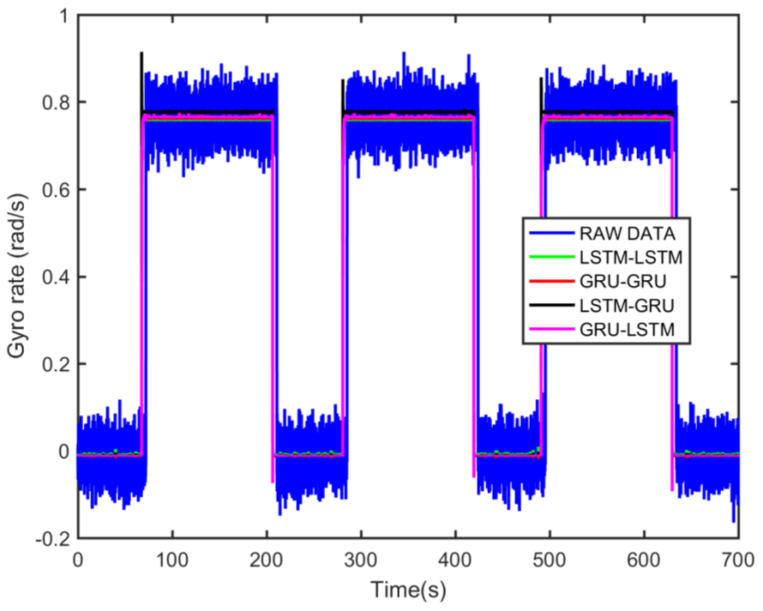
The gyro denoising results of four methods.

From static experimental results, it is clear that the proposed hybrid networks had good noise reduction effects, except GRU-LSTM. In order to further verify the effectiveness and applicability of the four RNN models, the actual dynamic signal from the gyro was acquired to experimentally verify the four models. The various parameters used therein were the same as explained in the static tests in Section 3.3. The dynamic tests are in the form of regular start and pause motions. The denoising results obtained with the different methods are presented in Figure 20. In addition, to further validate the effectiveness and variability of the proposed methods from both quantitative and qualitative perspectives, Hilbert spectral analysis and Allan variance were used to process the corresponding regular dynamic data in order to provide an intuitive and obvious comparison [55,56,57]. Hilbert spectra are used to statistically distinguish and resolve a mixture of moving signals. They are suited for nonlinear and non-smooth signal analyses. The process includes decomposing complex signals into a finite number of intrinsic mode functions, while the Hilbert-Huang spectrum is obtained by performing the Hilbert-Huang transformation on the intrinsic mode function generated before and after denoising the signals. The resulting instantaneous frequency variation with time shows the split-signal time-frequency energy distribution after the complex signal has been resolved.

**Figure 21:**
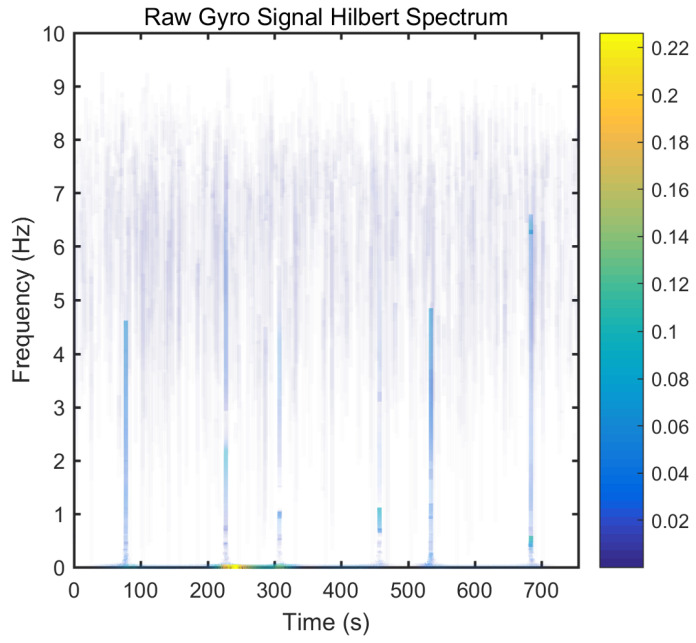
The Hilber spectrum results of the raw gyro signal.

**Figure 22:**
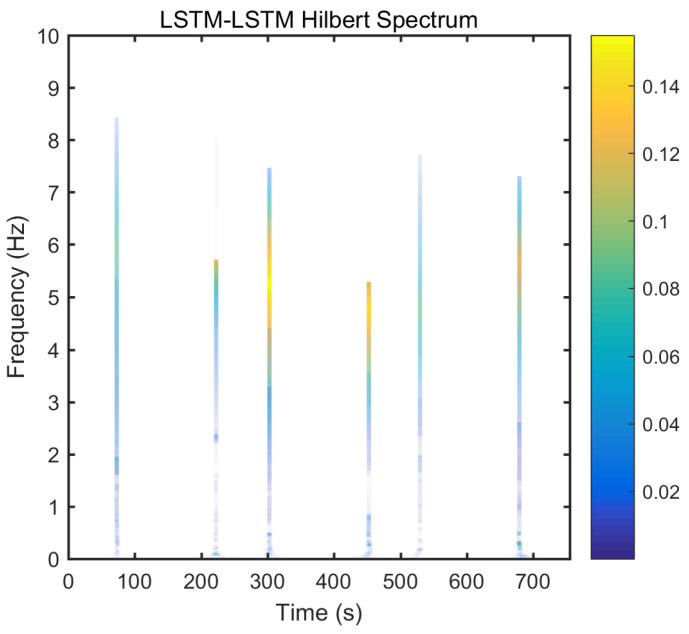
Hilber spectrum after LSTM-LSTM denoising.

**Figure 23:**
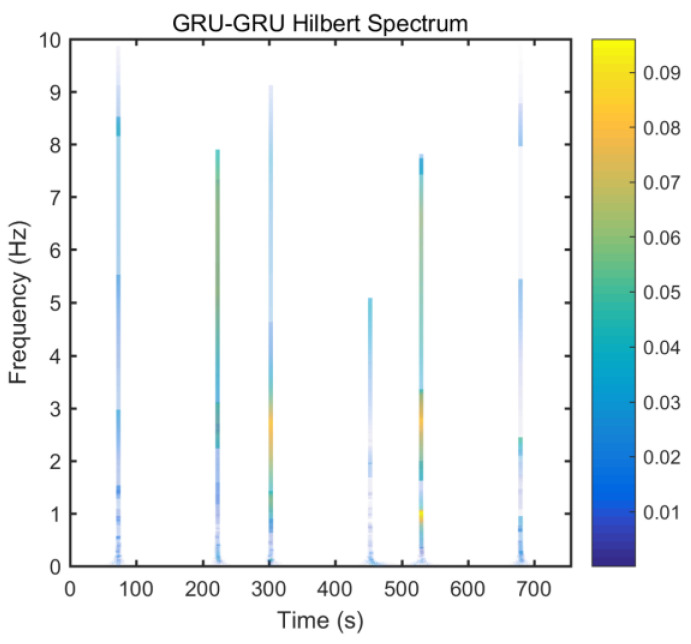
Hilber spectrum after GRU-GRU denoising.

**Figure 24:**
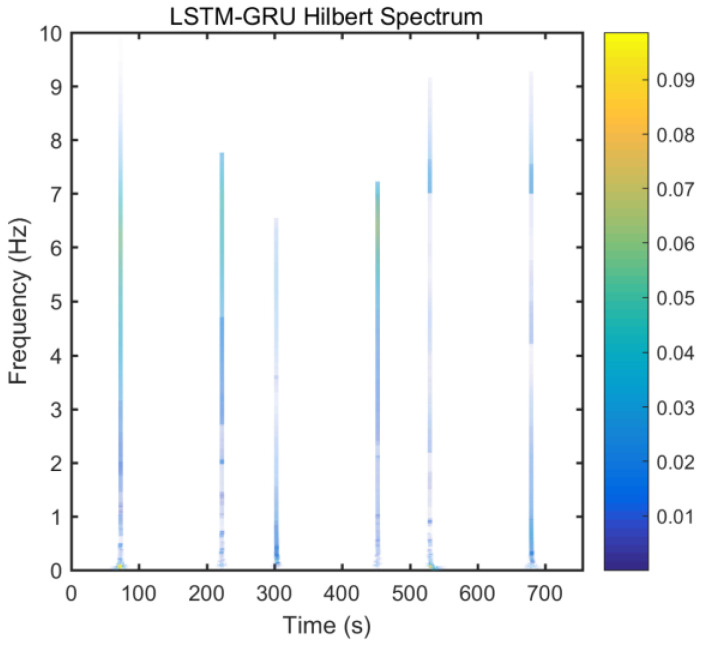
Hilber spectrum after LSTM-GRU denoising

**Figure 25:**
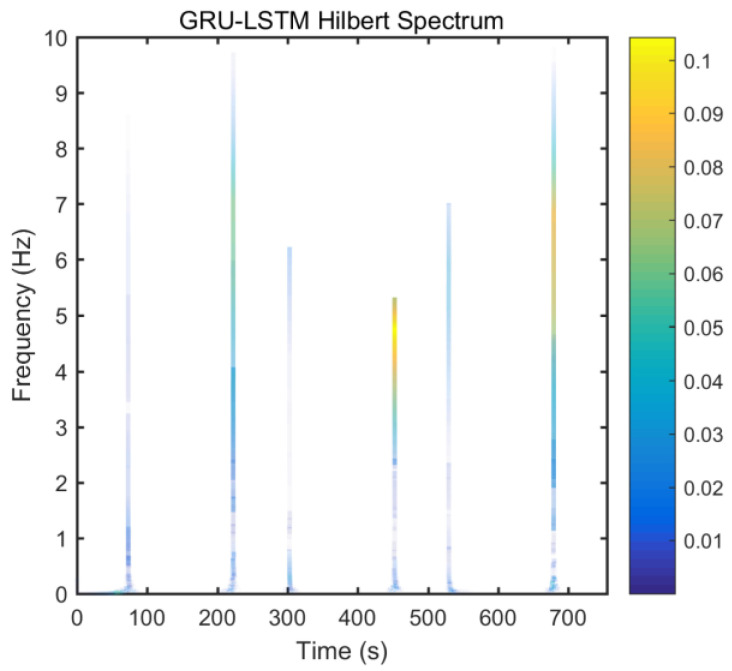
Hilber spectrum after GRU-LSTM denoising.

From Figure 21, it can be seen that many energy lines are concentrated in the middle and high frequency parts of the time–frequency domain, and these energy lines were relatively uniformly distributed throughout the time–frequency domain. Furthermore, it can be concluded that the high energy was mainly concentrated in the static dynamic rate transition and low frequency domain intervals, and the signal at the instant of static dynamic rate transition had significantly higher energy than the signal at static and dynamic rates. As shown in Figure 22, Figure 23, Figure 24 and Figure 25, the middle and high frequency noise components were almost completely removed after the denoising process by the different methods. Nevertheless, there was still some noise in the static and dynamic rate transition instant and the low frequency domain.

Since the useful information and noise are mixed together in the frequency domain bands between the static and dynamic rate transitions and the dynamic constant rate intervals, it is difficult or impossible to separate them very clearly. In addition, excessive noise cancellation may cause the useful information in this band to be removed along with the noise components thereby causing signal distortion. Aside from these reasons, the noise removal within the static and dynamic rate conversion and dynamic constant rate intervals is acceptable. As shown in Figure 22, Figure 23, Figure 24 and Figure 25, it can be seen that results of the time frequency energy distribution after denoising show that the applied models conformed to dynamics of the test motion form in the experimental data. This indicates that the denoised signals were not distorted. In addition, a comparative analysis of the denoising effects among the four algorithms shows that the time-frequency energies of the homogenous networks, i.e., the LSTM-LSTM and GRU-GRU along with that of the heterogeneous network GRU-LSTM were higher than those of LSTM-GRU in the whole frequency domain. It indicates that the denoising effect of LSTM-GRU was best. 

**Figure 26:**
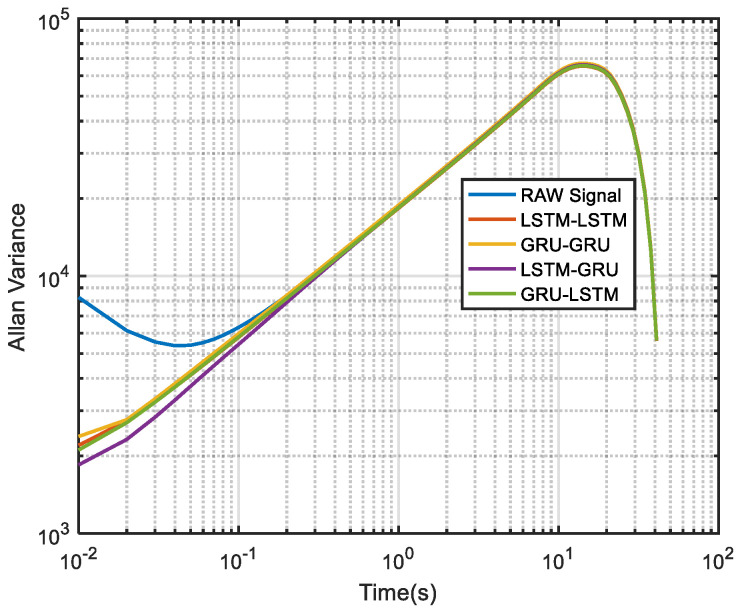
The corresponding Allan variance analysis of Figure 20.

To further demonstrate the denoising performance of the four recurrent networks in a quantitative sense, the Allan variance analysis of the original signal and the different denoised signals were plotted in Figure 26. Although Allan variance is the best analytical tool in the static evaluation of gyroscope, it can also be used to evaluate the time series dynamic signal, as can be confirmed in Refs. [56,57,58]. Comparing the Allan variance curves before and after denoising, it can be seen that there is a certain degree of decrease in the curves after the denoising with the four models. This means that a reduced signal-to-noise ratio was observed for the proposed methods, and in particular, the LSTM-GRU model shows the best performance. Table 5 records the execution times of the four denoising methods upon which the training results presented in Figure 20 was obtained. It can be seen from the table that GRU-GRU was the most time-efficient. This can mainly be attributed to the fact that GRU had fewer network structure parameters than an LSTM structure. Although all the four models had good noise reduction effects, especially LSTM-GRU, but their execution times were too long, and this could make them not to be very suitable for some practical applications. Therefore, further research is needed to shorten their running times for practical applications in this field.

## 4. Conclusions and Future Works

MEMS-IMU system is often affected by random noises which cause errors that seriously affect the navigation accuracy of MEMS-IMUs. Meanwhile, to improve the precision of MEMS-IMU and expand its application field, hybrid modes of DRNNs are developed for noise reduction of MEMS-IMU in this paper. Results and performances obtained for four methods namely, LSTM-LSTM, GRU-GRU, LSTM-GRU and GRU-LSTM are discussed. For this, we performed a test validation study using some sample dataset to determine the appropriate parameters for training the RNNs. The major parameters considered in this study were the training epoch, batch size, learning rate, amount of hidden units, and the time step. A consequence to this, quantitative evaluation of the denoising effects from the MEMS-IMU under the same conditions was done. The MEMS-IMU is a customized device developed in our lab, and it was used to perform the experimental studies with suitable data acquisition time of approximately 12 min. The results show that LSTM-LSTM, GRU-GRU, and LSTM-GRU all exhibited good denoising effects in large sample data of static experiments, especially LSTM-GRU works best, while GRU-LSTM is only suitable for a small sample test. In the dynamic experiments, both Hilbert spectrum and Allan variance show that all four algorithms have some degree of noise reduction effect. In summary, the different experimental results fully demonstrate the effectiveness and applicability of the proposed deep recurrent networks in MEMS gyro noise reduction, especially LSTM-GRU is more suitable for application in the MEMS gyro field compared with the other three algorithms, but the execution time of the four algorithms is too long for practical applications at present.

Additionally, we suggest that there are still further areas that could be studied to accelerate the improvement of DRNNs for MEMS-IMU accuracy and practical engineering applications. For instance, data length in this study was restricted by available computing power. Thus, the lengths of experimental data were not long enough and this should be considered in the presence of GPU acceleration. It is envisaged that longer experimental data will have a better improvement on the training and prediction accuracy of the hybrid deep learning models. Application of the models for real navigation trajectories is also vital. This could be used to further improve the models’ test performances for cases where MEMS-IMU devices are used for autonomous navigation such as in self-driving cars. In these areas, more multilayer hybrid structures of RNNs can be implemented and compared with existing models to enhance its application for time series data. For practical navigation, the convergence speed and tracking speed are very important since most of the motion forms are multirate motion while the direction and amplitude characteristics of the signals changes constantly. Therefore, rapid convergence and tracking in dynamic trajectory should also be studied.

## Figures and Tables

**Table 1:** Allan variance results of the *X* axis gyro.

Error Sources	*X*-axis Gyroscope				
	Raw Signal	LSTM-LSTM	GRU-GRU	LSTM-GRU	GRU-LSTM
Quantization Noise (deg/√h)	6.168649	2.577573	3.188908	0.331600	0.000211
Angle Random Walk (deg/√h)	12.774534	0.824106	0.976997	0.169090	0.000088
Bias Instability (deg/*h*)	190.759177	68.704943	113.726482	17.648027	0.028237

**Table 2:** Allan variance results of the *Y* axis gyro.

Error Sources	*Y*-axis Gyroscope				
	Raw Signal	LSTM-LSTM	GRU-GRU	LSTM-GRU	GRU-LSTM
Quantization Noise (deg/√h)	0.661380	0.149668	0.811900	0.057652	0.000385
Angle Random Walk (deg/√h)	1.748556	0.065753	0.308072	0.021857	0.000123
Bias Instability (deg/*h*)	68.970485	24.105904	36.667479	3.487848	0.017607

**Table 3:** Allan variance results of the *Z* axis gyro.

Error Sources	*Z*-axis Gyroscope				
	Raw Signal	LSTM-LSTM	GRU-GRU	LSTM-GRU	GRU-LSTM
Quantization Noise (deg/√h)	0.223371	0.021935	0.127073	0.009640	0.000463
Angle Random Walk (deg/√h)	1.445421	0.005576	0.037413	0.003515	0.000446
Bias Instability (deg/*h*)	33.720574	0.172867	9.586479	0.518550	0.053445

**Table 4:** Standard deviation of raw signal and denoised signals for a small sample (Figure 18).

Raw Signal	LSTM-LSTM	GRU-GRU	LSTM-GRU	GRU-LSTM
0.038767	0.001571	0.000959	0.000858	0.000623

**Table 5:** The execution time among different denoising methods (Figure 20).

Methods	Time (s)
LSTM-LSTM	490.83452
GRU-GRU	420.30138
LSTM-GRU	453.63127
GRU-LSTM	442.57631
